# The Flagellar Arginine Kinase in *Trypanosoma brucei* Is Important for Infection in Tsetse Flies

**DOI:** 10.1371/journal.pone.0133676

**Published:** 2015-07-28

**Authors:** Cher-Pheng Ooi, Brice Rotureau, Simonetta Gribaldo, Christina Georgikou, Daria Julkowska, Thierry Blisnick, Sylvie Perrot, Ines Subota, Philippe Bastin

**Affiliations:** 1 Trypanosome Cell Biology Unit, INSERM U1201, Institut Pasteur, 25 Rue du Docteur Roux, 75015, Paris, France; 2 Molecular Biology of Gene in Extremophiles Unit, Department of Microbiology, Institut Pasteur, 25 rue du Docteur Roux, 75015, Paris, France; University of Hull, UNITED KINGDOM

## Abstract

African trypanosomes are flagellated parasites that cause sleeping sickness. Parasites are transmitted from one mammalian host to another by the bite of a tsetse fly. *Trypanosoma brucei* possesses three different genes for arginine kinase (AK) including one (AK3) that encodes a protein localised to the flagellum. AK3 is characterised by the presence of a unique amino-terminal insertion that specifies flagellar targeting. We show here a phylogenetic analysis revealing that flagellar AK arose in two independent duplication events in *T*. *brucei* and *T*. *congolense*, the two species of African trypanosomes that infect the tsetse midgut. In *T*. *brucei*, AK3 is detected in all stages of parasite development in the fly (in the midgut and in the salivary glands) as well as in bloodstream cells, but with predominance at insect stages. Genetic knockout leads to a slight reduction in motility and impairs parasite infectivity towards tsetse flies in single and competition experiments, both phenotypes being reverted upon expression of an epitope-tagged version of AK3. We speculate that this flagellar arginine kinase is important for *T*. *brucei* infection of tsetse, especially in the context of mixed infections and that its flagellar targeting relies on a system equivalent to that discovered for calflagins, a family of trypanosome flagellum calcium binding proteins.

## Introduction

Trypanosomes are the causative parasites of human African trypanosomiasis, also called sleeping sickness. Although on the decline [1,2], the human and animal forms of the disease still constitute a major health and economic burden on the African continent. Transmission of the parasite relies almost exclusively on the infective bite of the tsetse fly (*Glossina* sp.). Therefore understanding the factors that allow for the successful infection of the insect vector remains a relevant research interest. The fly is more than a passive vector as the parasites undergo specific development programmes before becoming infective again. These can be relatively simple as in the case of *Trypanosoma vivax* that is mostly found in the mouthparts and alternates between two major forms. However, this is much more sophisticated in the *Trypanosoma brucei* subgroup (*T*. *b*. *brucei*, *T*. *b*. *rhodesiense* and *T*. *b*. *gambiense*), where infection is established in the posterior midgut and proceeds anteriorly towards the proventriculus to culminate in the salivary glands, giving rise to metacyclic trypomastigotes that are the only parasites infective to mammalian hosts [3,4]. An intermediate situation is encountered in *T*. *congolense* that proliferates first in the gut and then reaches the proboscis before attaching to the surface of the labrum and releasing infective cells in the hypopharynx [5]. To survive in all these different environments, trypanosomes adapt their shape and morphology, their metabolism and their surface protein composition [6].

Trypanosomes are members of the Excavata, one of the major early branching eukaryotic lineages. Their morphology is characterised by an elongated shape and the presence of a flagellum attached to the cell body. The flagellum is a multifunctional organelle, with known roles in motility, morphogenesis and attachment to the epithelium of the salivary glands (review in [7]). It has also been proposed to function in environmental sensing [8–11]. Biophysical analysis demonstrated that flagellum disposition and motility are particularly well suited to swimming in a crowded environment among erythrocytes, such as encountered for the bloodstream form of the parasite [12]. Recently, forward flagellum-mediated motility has been shown to be crucial for parasites to migrate anteriorly from the posterior midgut to the anterior midgut of the tsetse [13]. These data raise the question of the control of flagellum motility and how it could be modulated to fit the different environments encountered by *T*. *brucei* during its cyclical development.

A proteomics analysis of intact flagella purified from cultured procyclic trypanosomes revealed the presence of the enzyme arginine kinase (AK) in the membrane and matrix fraction [10]. The enzyme shows rapid turnover in both mature and growing flagella but RNAi silencing suggests it is not required for growth in culture [10]. The flagellar location has also been confirmed upon epitope tagging and overexpression in cultured procyclic *T*. *brucei* [14]. The presence of this enzyme captured our attention because it belongs to the phosphagen kinases, enzymes that reversibly dephosphorylate ATP and phosphorylate a specific substrate, forming an ATP buffer system for the temporally rapid but limited regeneration of ATP when required (reviewed in [15]). In both spermatozoa and protists, flagellar beat frequency relies on the presence of ATP. In the flagellum of spermatozoa, phosphagen kinases are thought to act as energy shuttles to transfer phosphates from their point of synthesis to the beating flagellum where ATP consumption occurs [16,17]. While evidence is not extensive regarding the same function in flagellated protists, exposure of *Paramecium caudatum* cilia to ADP decreases beat frequency, but is restored upon addition of phosphorylated arginine [18]. This argues that although direct evidence is lacking for phosphagen kinases as a *bona fide* energy shuttle in flagellated protists, generation and access to phosphagens could be important in maintaining the beat frequency of spermatozoa and protist flagella.

Here we investigated the possible role of arginine kinase during parasite development in the tsetse fly. We present phylogenetic data revealing that trypanosome *AK* genes were likely acquired by horizontal gene transfer from an invertebrate host and that separate duplication events led to the emergence of flagellar forms of AK in *T*. *brucei* and possibly in *T*. *congolense*, the two species that undergo the most exhaustive development in the tsetse fly. The flagellar AK is expressed in all stages of development but it appears more abundant in insect stages compared to the bloodstream form. Knockout of the flagellar *AK* in *T*. *brucei* mildly affects parasite motility but leads to lowered infectivity and competitiveness in tsetse, supporting a contribution to infection. To our knowledge, this is the first *in vivo* evidence regarding the possible relevance of flagellar AK in the life cycle of *T*. *brucei*. Finally, we speculate about the possible role of AK3 in trypanosome development and about its mechanism of flagellar targeting.

## Materials and Methods

### Ethics statement

This study was carried out in strict accordance with the recommendations in the Guide for the Care and Use of Laboratory Animals of the European Union (European Directive 2010/63/UE) and the French Government. The protocol was approved by the “Comité d’éthique en expérimentation animale (CETEA) de l’Institut Pasteur” N°89 (Permit Number: 2013–0128 Blisnick). All efforts were made to minimize suffering.

### Phylogenetic analysis

Homologues of TbAK were exhaustively searched by Blastp in current sequence databases. Multiple protein alignments were performed with Muscle [19], and trimmed with BMGE [20]. Based on preliminary phylogenetic trees, a selection of 62 representative taxa was chosen for more detailed analysis. Bayesian analysis was carried out on a dataset comprising 220 unambiguously aligned amino acid positions by PhyloBayes, with the LG model and 4 categories of evolutionary rates [21]. Two MCMC chains were run in parallel until convergence, and the consensus tree was calculated by removing the first 25% of trees as burnin. In order to clarify the gene duplications leading to the paralogous *AK* copies in *T*. *brucei* and *T*. *congolense*, further analysis was restricted to the trypanosome only sequences, and performed on nucleotides. A maximum likelihood tree was obtained by PhyML, the GTR model and 100 boostrap replicates of the original alignment.

### Culturing of parasites

Cell lines for the investigation were generated from the tsetse-transmissible *T*. *b*. *brucei* AnTat 1.1 (wild-type or WT) cell line [22]. Procyclic trypomastigotes of the WT cell line and its derivatives were cultured in SDM-79 [23] supplemented with 10% foetal bovine serum and 20 mM glycerol [24](SDMG for SDM-79 + Glycerol). Cell concentration was determined using the Z2 cell counter (Beckman Coulter). Bloodstream AnTat 1.1 trypanosomes were cultured in Creek’s minimum medium [25] at 37°C, 5% CO_2_ and allowed to attain 2 X 10^6^ parasites/ml culture prior to harvest for transformation to the procyclic stage, IFA or western blots for the comparison of AK3 amounts between bloodstream and procyclic trypanosomes. For transformation, bloodstream trypanosomes were suspended in DTM medium with 3 mM isocitrate/*cis*-aconitate and cultured at 27°C for 24 h [26]. Transformed parasites were subsequently passaged into SDMG medium. Drug selection was always removed prior to generating growth curves. Cells were spun down, resuspended in medium without drugs prior to measuring growth over time. Cultures were maintained in medium without drugs throughout the duration of the experiment. The only exception for this was for the inducible cell line, where tetracycline was added to the induced culture after re-suspension in medium.

### Generation of cell lines

For the purpose of generating a cell line deprived of AK3, we used a double knockout approach. Sequences containing either the puromycin (*PURO*) or the blasticidin (*BLA*) drug resistance gene flanked by upstream and downstream regions of the *AK3* gene (Tb927.9.6170, 300 bp long each) were chemically synthesized and cloned in a pUC57 plasmid (Genecust Europe, Luxembourg). PCR amplicons were generated with primers F1: TTTGGCAAATGCTCACAAGA and R1: TCTATACACAAGTACCTCCC (Eurogentec) which anneal to the sequences 300 bp upstream of the *AK3* start codon and downstream of *AK3* stop codon, respectively. Amplicons with either the blasticidin resistance gene (999 bp) or the puromycin resistance gene (1,200 bp) were gel-purified (Macherey-Nagel) prior to transfection. Nucleofections were carried out using the Amaxa nucleofactor (Lonza)(Burkard et al MBP2007) and clonal populations were obtained with sub-cloning by limiting dilution. Generation of the ∆*ak3* cell line was accomplished by 2 successive rounds of nucleofection to replace both alleles of endogenous *AK3* in WT with *BLA* and *PURO* genes. For the purpose of generating an add-back cell line (AB), the *AK3*::*Ty1* gene was synthesized and integrated into the pHD430 plasmid [27] by Genecust Europe to generate the pHD430AK3::Ty1 plasmid. The sequence contains the first 1125 nucleotides of *AK3*, the 30 nucleotides encoding the Ty1 sequence (GAGGTCCATACTAACCAGGACCCACTTGAC)[28] and the last 90 nucleotides of *AK3* including the stop codon. This plasmid was linearized with the NotI restriction enzyme prior to nucleofection for integration into the ribosomal intergenic spacer in the ∆*ak3* cell line. An additional round of nucleofection was carried out on clonal AB cell line with the pHD360 vector [27] that was targeted to the tubulin locus to ensure expression of tet-repressor, hence rendering the production of the AK3::Ty1 protein tetracycline-inducible [29]. As the generated cell lines were to be used for tsetse infection experiments, only one clonal cell line from each was chosen for further analyses. To confirm integration of the AK3 replacement cassettes and the presence or absence of *AK3* in the genome of the transformed cells, genomic DNA was isolated [30] and PCR was performed using primers F1 and R1 (see above) or F2: CACGGCTAATCCTGTTTCTA and R2: GAAAAAGAAAGGGGAGGGCA that annealed upon the transcribed region of *AK3*.

### Maintenance and infection of tsetse

Teneral males were collected 24 to 48 h post-eclosion in our tsetse insectarium that is maintained at 27°C and 70% hygrometry. Collected flies were separated into Roubaud cages and infected by directly feeding with SDMG medium containing 6–9 X 10^6^ parasites/ml (either with single cell lines or with equal mix of 2 cell lines) as a first meal through a silicone membrane. Flies were fed twice a week with defibrinated sheep’s blood and starved for at least 48 h prior to dissection. Dissections were carried out on glass slides in PBS under a binocular dissection microscope by removing the alimentary canal of the tsetse from the hindgut up to the crop and proventriculus to determine the presence or absence of parasites in the various sections (hindgut, posterior midgut and anterior midgut). Salivary glands were dissected in a separate drop of PBS on the glass slide. Infection data from dissection experiments were only considered for statistical analysis in biological replicates where more than 10 individual insects were left alive in all experimental groups at the time of dissection. For parasites meant for IFA analyses, the whole gut was torn apart with forceps and the parasites were transferred into an Eppendorf on ice, while individual sections of the alimentary canal were removed and dropped directly into separate Eppendorf tubes with 100 μl of PBS on ice when checking for the proportion of cells expressing AK3::Ty1. Both samples were subsequently treated with 4% PFA prior to IFA.

### Immunofluorescence assays

Sample preparation for immunofluorescence assays (IFA) was as described in [31]. Briefly, tsetse harvested trypanosomes were fixed in 4% PFA and allowed to settle on poly-L-lysine coated slides. Cultured parasites were washed once with serum-free medium and suspended in 4% paraformaldehyde prior to centrifugation onto poly-L-lysine coated slides with the cytospin system (Thermo Scientific). Adhered cells were washed in PBS between each step in the protocol. Adhered cells were then permeabilised with 0.1% Nonidet P-40 in PBS for 5 min, blocked with ammonium chloride (60 mM in PBS) for 10 min and subsequently blocked with 0.1% bovine serum albumin in PBS for 45 min at room temperature [31]. Alternatively, parasites extracted from flies were fixed for 30 seconds in methanol at -20°C and rehydrated for 10 minutes in PBS. Adhered cells were subsequently probed with the following primary antibodies diluted in 0.1% bovine serum albumin in PBS. Two different batches of anti-TcAK were used: the first one was kindly provided by Claudio Pereira (CONICET, Buenos Aires, Argentina) [32] and was used for the initial experiments, including the original characterisation of AK3 [10]. It will be referred to as the Buenos Aires anti-TcAK. The second one was generated in-house at the Institut Pasteur by inoculation of mice with recombinant TcAK, expressed from the vector provided by C. Pereira [33]. It will be termed the Paris anti-TcAK. To detect the Ty1 epitope, either the monoclonal antibody BB2 [28] (diluted at 1:200) or a rabbit anti-Ty1 (1 μg/ml, GenScript) were used. Fluorophore-linked (Cy3, Dylight 488) subclass-specific secondary antibodies raised against mouse or rabbit (Jackson ImmunoResearch) were used at 1:250 dilution to visualize primary antibodies. Antibody probing was carried out for 1 h at 37°C within a humid chamber. Cells were finally stained with DAPI (1 μg/ml) for 1 min and treated with Prolong anti-fading agent (Invitrogen) before being sealed with a coverslip.

IFA Image acquisition was carried out on a Leica 4000B microscope with a 100 X objective lens using a Hamamatsu ORCA-03G camera. The fluorescence light source was from a Lumencore LED system with the following incident light intensities: Cyan (5%, Dylight-488), UV (50%, DAPI) and Green (2–5%, Cy3). Image acquisition was controlled using Micro-manager and images were taken with the min/max threshold set at maximum. Subsequent normalization of signals was carried out by parallel manipulation of min/max signal against controls in ImageJ [34], and images were superimposed using Photoshop CS4.

### Western blotting

For western blotting, sampled were prepared by washing cells in serum-free medium before dilution with PBS and boiling in Laemmli. SDS-PAGE separation was carried out after loading 10 μg (corresponding to 1 million cells) total cell protein per lane. Proteins were transferred onto polyvinylidene fluoride membranes overnight at 4°C and blocked with 5% skimmed milk in PBS with 0.1% Tween 20 for 1 h prior to probing. The following primary antibodies were used by dilution in 1% milk in PBS with 0.1% Tween 20: L8C4 [35] (diluted 1:20), (di-TcAK [32](or produced as described above)(diluted 1:5,000) and BB2 [28] (undiluted). Anti-mouse and anti-rabbit horseradish peroxidase conjugate secondary antibodies (Amersham Biosciences) were used at 1:20,000 dilution. Membranes were washed in PBS with 0.1% Tween 20 after blocking, between probing with primary and secondary antibodies and prior to band detection by chemiluminescence (GE healthcare). Band analysis was carried out with ImageJ64 [34] and images were converted to greyscale and contrasted using Adobe Photoshop CS4.

### TEM analysis

Cell samples were first fixed in 2.5% of glutaraldehyde directly in culture medium and then in 2.5% glutaraldehyde, 4% PFA in 0.1M cacodylate buffer overnight at 4°C. Cells were then postfixed in 1% osmium tetroxide, uranyl acetate and after serial dehydration, embedded in epon and polymerized at 60°C. Ultrathin sections are collected on copper grids and stained with uranyl acetate and lead citrate. Observations are made on a Tecnai10 electron microscope with MegaView II camera and processed with AnalySIS and Adobe Photoshop CS4 (San Jose, CA)[36].

### Motility analysis

Cells at 6–9 X 10^6^ were maintained in SDMG medium at 27°C prior to video acquisition. Movies (20 s duration, 100 ms exposure, 176 frames) were captured with a 10 X objective lens on a Leica 4000B microscope with a Hamamatsu ORCA-03G camera using HCImage Live software (Hamamatsu). Movie capture was carried out in an enclosed room maintained at 25°C. Videos were converted to MPEG format and analysed using medeaLab CASA Tracking V.5.5 software (medea ABGmbH).

### Statistical analysis

One-way ANOVA, non-continuous t-tests, linear regression were carried out on Prism (GraphPad). Differences are considered significant if P < 0.05 (denoted with *) and highly significant if P < 0.001 (denoted with **).

## Results

### Origin and expansion of *AK* genes in trypanosomatids

**Fig 1:**
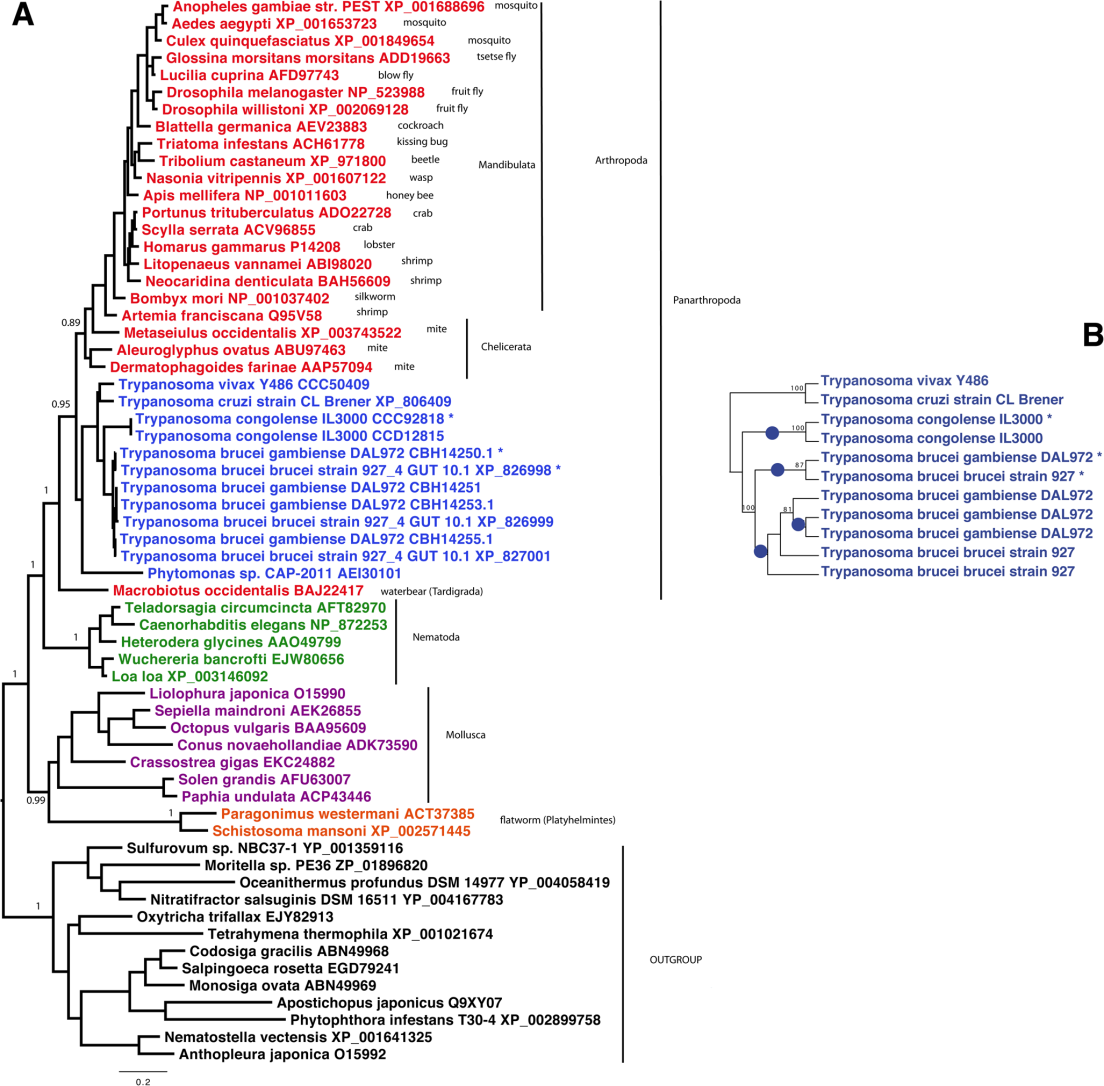
Phylogenetic analysis of arginine kinases. A. Bayesian tree of AK homologues from 62 representative taxa. Trypanosome AK3 homologues are indicated by asterisks. The tree was calculated by PhyloBayes, with the LG model and four categories of evolutionary rates. Two MCMC chains were run in parallel until convergence, and the consensus tree was calculated by removing the first 25% of trees as burnin. For clarity, posterior probabilities supports are shown for the most important nodes only. The scale bar represents the average number of substitution per site. B. Maximum likelihood tree obtained from trypanosome only sequences obtained by PhyML with the GTR model. Blue dots represent the inferred species-specific independent gene duplications leading to specialized AK homologues. Values at nodes represent support obtained by nonparametric bootstrapping from 100 replicates of the original alignment. In order to clarify the gene duplications leading to the paralogous *AK* copies in *T*. *brucei* and *T*. *congolense*, further analysis was restricted to the trypanosome only sequences, and performed on nucleotides. A maximum likelihood tree was obtained by PhyML, the GTR model and 100 bootstrap replicates of the original alignment.

Genes encoding AK are mostly encountered in arthropods, nematodes, molluscs and flatworms (Fig 1). The first report for AK in trypanosomatids was published by Pereira and co-workers who cloned an *AK* gene from *Trypanosoma cruzi*, highlighting the remarkable conservation of the core protein that shows ~70% identity with various insect or crustacean AK [33]. Since then, genome sequencing has shown the presence of *AK* genes in all *Trypanosoma* subspecies investigated to date but not in *Leishmania* [32]. Intriguingly, different number of *AK* genes were identified according to the species: 4 in *T*. *b*. *gambiense*, 3 in *T*. *brucei* and *T*. *evansi*, 2 in *T*. *congolense* and only 1 in *T*. *vivax* or *T*. *cruzi* (Table 1). The *AK* genes are located on chromosome 9 of the *T*. *brucei* genome in a tandem arrangement. Previous authors have differed in their nomenclature for the 3 AK versions in *T*. *brucei* [14,32,37]. In this publication we follow the rule of precedence by referring to the flagellar-targeting AK as AK3, the glycosomal one AK2 and the cytosolic one as AK1 [14][32]. AK1 consists of a core arginine kinase protein while AK2 has a peroxisome targeting sequence on its C-terminus and AK3 has two insertions of respectively 22 and 26 amino acids at the N- and C-terminus. Overexpression of full-length and truncated versions of epitope tagged AK3 indicates that the 22 N-terminal amino acids are responsible for flagellum targeting [14] (see below).

**Table 1:** List of *AK* genes in trypanosomatids.

	Gene name	aa number	pI	N-terminal tail
*T*. *brucei*				
Tb927.9.6290	*AK1* *	356	6.4	-
Tb927.9.6230	*AK2* *	370	5.9	-
Tb927.9.6170	*AK3* *	404	4.9	+
*T*. *gambiense*				
Tbg972.9.3300	*AK1*	356	6.5	-
Tbg972.9.3280	*AK2*	370	6.2	-
Tbg972.9.3260	*AK2*	370	6.3	-
Tbg972.9.3250	*AK3*	404	4.9	+
*T*. *evansi*				
TevSTIB805.9.4500	*AK1*	356	6.4	-
TevSTIB805.9.4480	*AK2*	367	6.1	-
TevSTIB805.9.4470	*AK3*	404	5.1	+
*T*. *congolense*				
TcIL3000.0.36630	*AK1*	356	5.9	-
TcIL3000.9.2150	*AK3*	372	6.4	+
*T*. *vivax*				
TvY486.0902320	*AK2*	375	6.6	-
*T*. *cruzi*				
TcCLB.507241.30	*AK1*	357	6.7	-

* Here the original nomenclature [32] was followed. These genes have been termed TbAK3 (AK1) and TbAK1 (AK3) by [14].

Phylogenetic analysis of AK homologues is presented in Fig 1A where AK3 homologues are indicated by asterisks. *Trypanosoma* AK proteins form a robustly supported monophyletic group with *Phytomonas*, indicating presence of an *AK* gene in the ancestor of this clade. Consistently with previous findings [37], this group is solidly embedded within the basal radiation of Panarthropoda, suggesting that it was very likely acquired via horizontal gene transfer from an early lineage of this metazoan clade, which was already diversified by the Cambrian (around 500 million years ago). Remarkably, the three *AK* copies present in *T*.*brucei brucei* and *T*.*brucei gambiense* are more closely related among them than they are to those of their kins, and the same is true for the two AK copies present in *T*.*congolense*. This indicates that the flagellar AK arose twice independently in *T*. *brucei* and *T*. *congolense*, possibly as an adaptation to their development in the tsetse midgut. A more detailed analysis based on the *Trypanosoma* AK nucleotidic sequences shows more clearly these parallel events (blue dots in Fig 1B).

**Fig 2:**
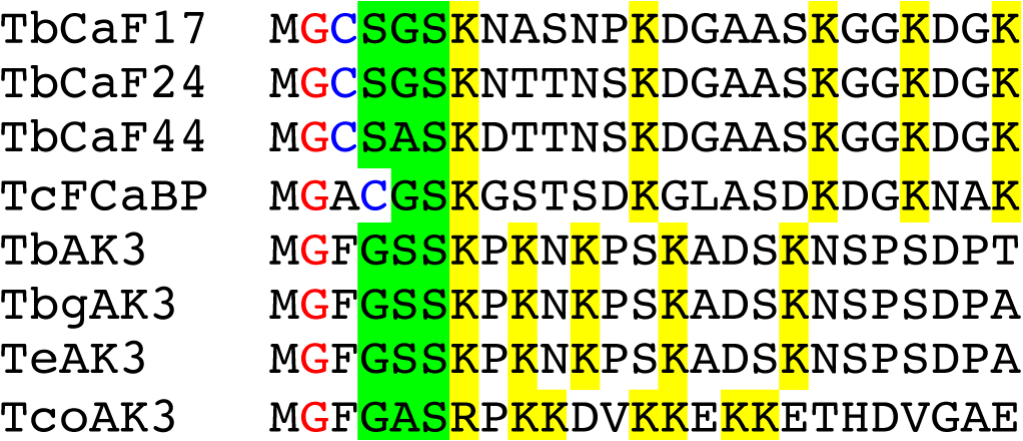
The flagellar targeting sequence of AK3 shares close similarity with that of calflagins. Alignment of the first 25 amino acids of *T*. *brucei* calflagins, the *T*. *cruzi* FCaBP and AK3 from *T*. *brucei*, *T*. *b*. *gambiense*, *T*. *evansi* and *T*. *congolense* with conserved residues indicated in colour. The glycine and the cysteine used for myristoylation and palmitoylation of calflagins are shown in red and blue, respectively. The lysine residues are highlighted in yellow while short amino acids (serine, glycine, alanine) always found in positions 4–6 are highlighted in green.

In their targeting studies, Voncken et al. could not identify a conserved domain that would explain the targeting of AK3 into the flagellum [14]. Here, we noticed an amazing similarity between the short N-terminal sequence of AK3 and the one reported to be essential for flagellar targeting of a family of calcium-binding proteins: FCaBP in *T*. *cruzi* and the calflagins in *T*. *brucei*. These acidic proteins (pI ~4.5) possess EF-hands and an amino-terminal tail of 24 amino acids that is necessary and sufficient for flagellum location [38,39]. This sequence contains several key residues required for flagellum targeting including a glycine in position 2 and a stretch of positively charged amino acids (usually lysines) [38,40,41]. Analysis of the amino-terminal composition of available AK3 sequences revealed striking common features (Fig 2): the occurrence of a glycine in position 2 and the presence of 5 lysine residues, exactly as in FCaBP and in all 3 calflagins. Moreover, 3 small residues (glycine, serine or alanine) are always present in flagellar AK and in calflagins in position 4 to 6. We also note that AK3 is acidic with a predicted pI of 4.9 instead of 5.9 and 6.2 for AK2 and AK1, respectively. The longest *T*. *congolense* AK protein recapitulates most of these features: amino-terminal positioning, glycine in position 2 and 7 positively charged residues (including 6 lysines).

### Flagellar localisation of AK3 persists throughout the *T*. *brucei* life cycle

**Fig 3:**
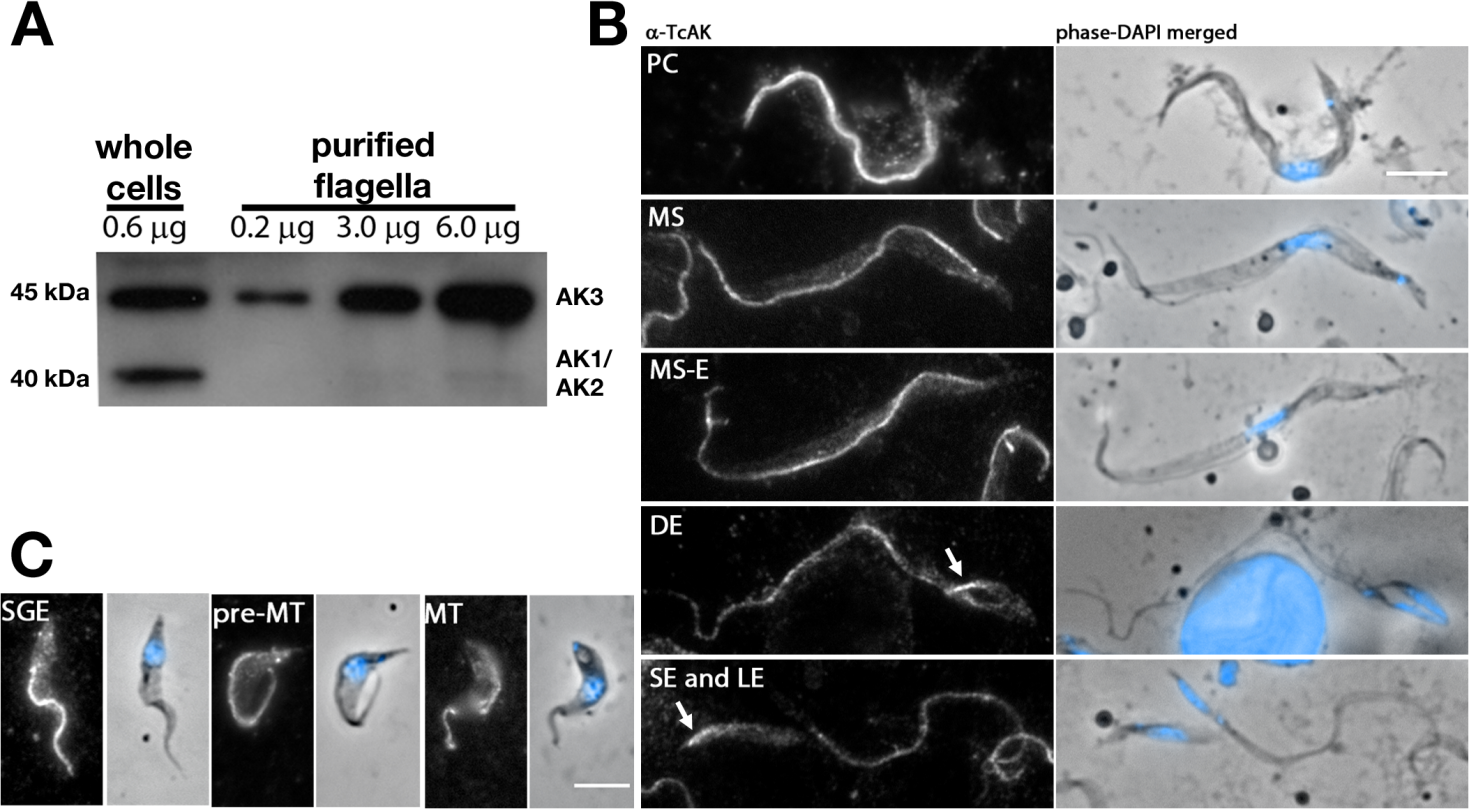
Endogenous AK3 localises to the trypanosome flagellum in all stages of the life cycle. A. Immunoblotting of whole cell lysate and flagellar samples from induced *FLA1* ^*RNAi*^ cells with the original (Buenos Aires) anti-TcAK antiserum reveals AK3 to be specifically enriched within the parasite flagellum. B,C. IFA using the same anti-TcAK antiserum diluted 1:100 on AnTat 1.1 cells extracted from infected tsetse flies revealing persistence of the flagellar signal in all life cycle stages of *T*. *brucei* in the fly. This includes forms encountered in the digestive track (B) such as the procyclic (PC), mesocyclic (MS), mesocyclic to epimastigote transition (MS-E), dividing epimastigote (DE) and epimastigote (both long, LE and short, SE), as well as those present in the salivary gland (C): salivary gland attached epimastigote (SGE), pre-metacyclic (pre-MT) and metacyclic (MT). Arrows point at the flagellum of short epimastigote cells that was often more brightly stained than the long flagellum. Scale bars are 5 μm.

During the proteomic analysis of intact flagella, all AK peptides were derived from the conserved central core of the protein [10]. Hence, it was not possible to discriminate from which of the 3 AK they were derived. We therefore analysed samples of intact flagella and whole cells from the *FLA1* ^*RNAi*^ strain [10] by western blotting using the original antiserum raised against the single *T*. *cruzi* AK protein [33]. This antibody stains the membrane of flagellum in procyclic *T*. *brucei* by immunofluorescence assay (IFA), a signal that is lost upon AK3 RNAi silencing (Subota et al MCP2014). In total protein samples (Fig 3A), two bands were clearly visible, one migrating close to the 40 kDa marker, a size compatible with AK1 (predicted mass 40.1 kDa) and AK2 (41.6 kDa). In some westerns, this band could potentially be resolved as a doublet of closely migrating proteins (Fig 2A). These bands likely correspond to AK1 and AK2, although formal demonstration would need direct investigation of these two proteins. The other band is close to the 45 kDa marker and could match with AK3 whose predicted molecular weight is 44.7 kDa. Only this band was detected in samples of intact flagellum (Fig 3A), suggesting that AK3 is the only AK to be localised in the flagellum. This fractionation experiment is compatible with the overexpression of a tagged version of AK3 [14] and confirms that the endogenous AK3 indeed localises to the flagellum of cultured procyclic *T*. *brucei*.

The anti-TcAK antibody was used to investigate the localisation and expression level of AK in the developmental stages of trypanosomes found in the tsetse fly (Fig 3B and 3C). *G*. *morsitans morsitans* were infected with the AnTat1.1 strain and dissected at different time points. In order to obtain a sufficient high number of parasites, we used methanol fixation before performing IFA with the anti-TcAK antibody. This fixation method allows retention of much larger numbers of parasites on the slide compared to aldehyde fixations. In all trypanosome cells issued from the alimentary tract (procyclic, mesocyclic, mesocyclic differentiating to epimastigote, dividing epimastigote, long or short epimastigote, Fig 3B) and the salivary gland of the tsetse fly (attached epimastigote, pre-metacyclic and metacyclic, Fig 3C), the AK signal was weakly detected in the cytoplasm but strongly present in the flagellum (Fig 3). In contrast to ALBA proteins that are RNA-binding proteins involved in parasite development in the tsetse fly, the signal was not reduced in proventricular stages [42]. The AK signal in flagella of the short epimastigote form appeared stronger than in the long epimastigote form, analysed in the same field, before and after cell division (Fig 3).

**Fig 4:**
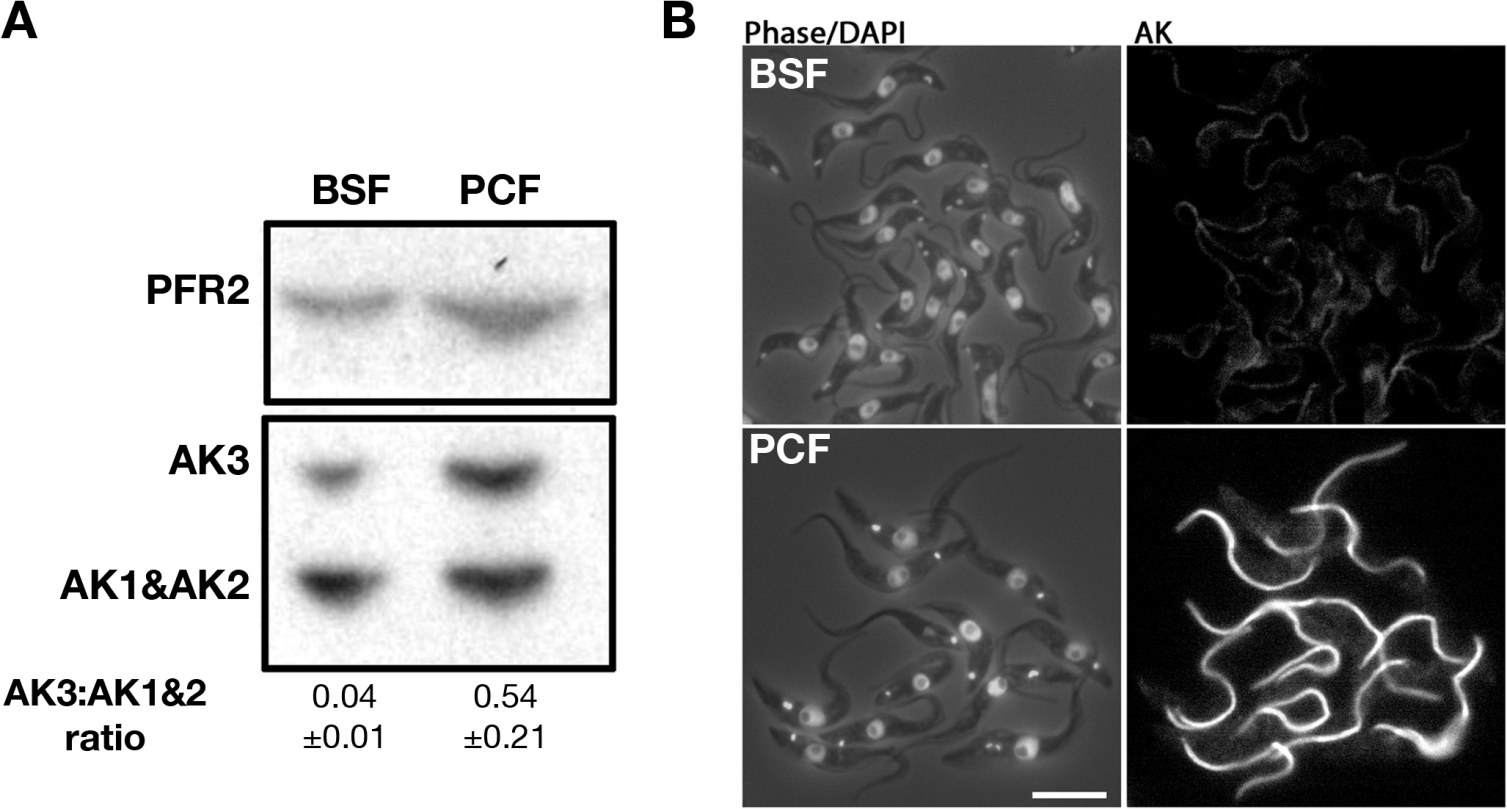
Expression of AK3 is more prominent in procyclic than in the bloodstream form. A. Immunoblotting of whole cell lysate with the Paris-raised anti-TcAK polyserum shows that AK3 is more highly expressed in tsetse-infective procyclic trypanosomes compared to bloodstream trypanosomes, as confirmed by the indicated AK3:(AK1+AK2) band ratio derived from mean band intensity of 3 independent experiments. B. IFA with the same antibody showing that the normalised signal of flagellar AK3 is lower in bloodstream cells than that in procyclic cells. The scale bar is at 10 μm.

Since the original mouse anti-TcAK antiserum had run out, we produced a fresh stock of antiserum by immunising mice with the TcAK expressed in *E*. *coli* from the same expression construct kindly provided by C. Pereira [33]. This new antibody was used in all subsequent experiments and yielded similar results as the original one (see below). To investigate the presence and distribution of AK in the *T*. *brucei* bloodstream form and in freshly transformed procyclic trypanosomes, western blot and IFA analyses were performed (Fig 4). As for the Buenos Aires antiserum, it detects two major bands corresponding to the expected molecular weight of AK3 and AK1/AK2. The relative level of AK3 was higher in procyclic trypanosomes compared to bloodstream trypanosomes, a finding confirmed in three separate experiments (Fig 4A). This observation was supported by IFA experiments where the flagellar signal was less pronounced in bloodstream trypanosomes compared to procyclic trypanosomes (Fig 4B). These data support the differential regulation observed at the mRNA level by northern blotting [14] and are in agreement with the detection of AK3 in the flagellum of bloodstream *FLA1* ^*RNAi*^ cell line [8]. Furthermore, IFA carried out on trypanosomes extracted from infected tsetse flies confirmed the flagellar-specific signal (see below) equivalent to when probed with the original anti-TcAK.

### Generation of cell lines for infection studies within tsetse flies

Several lines of evidence suggest that AK3 could perform more significant function(s) during parasite development in the fly: restricted presence in *T*. *brucei* and *T*. *congolense* that are the only species to develop in the midgut, higher expression level in procyclic cells versus bloodstream counterparts and expression in all development stages encountered in the tsetse fly. Generation of an RNAi inducible knockdown procyclic cell line in a 29–13 background where all 3 AKs were targeted simultaneously (*AK1–3* ^*RNAi*^) resulted in total loss of the flagellum signal, also confirming the specificity of the antibody [10]. Nevertheless, no pronounced growth phenotype could be observed [10]. This suggests that none of the AK are essential for growth in culture. However, RNAi never gives 100% silencing and possibly a reduced amount of AKs may be sufficient for cell viability. Yet another option is that AK3 may be important beyond the context of cultured parasites during fly infection.

**Fig 5:**
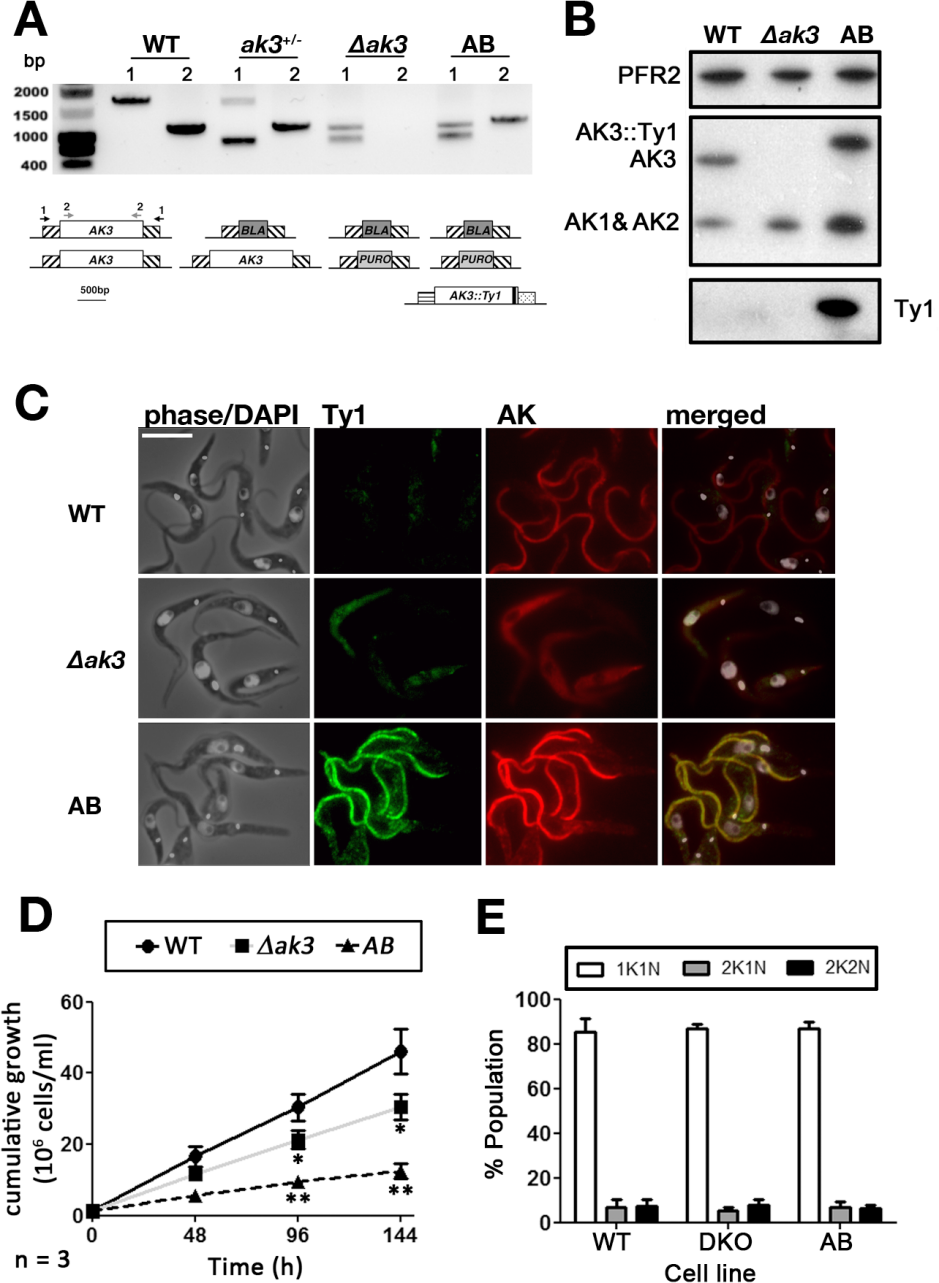
Generation of stable knockout (*∆ak3*) and add-back (AB) cell lines. A. PCR analysis using primers annealing to either the UTRs (1, F1 and R1 in materials and methods) of *AK3* or *AK3* itself (2, F2 and R2 in materials and methods) indicates that the locus is uninterrupted in WT cells, while the single knockout (KO) cell line has one *AK3* allele replaced by a smaller *BLA* resistance gene. The *∆ak3* knockout has had both *AK3* alleles replaced by a *BLA* resistance gene and a *PURO* resistance gene. The AB cell line retains both drug resistance markers, but has acquired the tagged copy of *AK3*. B. Immunoblotting with the Paris anti-TcAK antiserum reveals that the *∆ak3* cell line no longer expresses AK3 while AK3::Ty1 is expressed in the AB cell line. Both the *∆ak3* and AB cell lines express more AK1 and AK2 than WT cells while the AK3::Ty1 is more highly expressed in AB cells compared to AK3 in WT cells. C. IFA with the Paris anti-TcAK polyserum and anti-Ty1 antibody demonstrates that AK3::Ty1 in the AB cell line localises to the parasite flagellum. The scale bar is 10 μm. D. Cells that do not express AK3 (*∆ak3*) have a significantly lowered growth rate compared to WT cells. Growth rate is further slowed in AB cells expressing exogenous AK3::Ty1. E. DNA staining with DAPI revealed that there was no significant deviation from WT karyotype (n = 196) in the growth impaired *∆ak3* (n = 178) and AB (n = 193) cell lines.

To address these issues, two cell lines were generated from the AnTat 1.1 (WT for wild type) background, which is tsetse infective. First, a *∆ak3* cell line with both copies of *AK3* replaced by a puromycin and a blasticidin resistance gene was generated. Second, an exogenous Ty1-tagged version of *AK3* (*AK3*::*Ty1*) was added from a subsequently transfected pHD430 vector [27] into the *∆ak3* cell line, generating the *∆ak3 + AK3*::*Ty1* (AB for Add-Back) cell line that constitutively over-expresses TbAK3::Ty1 from the rDNA spacer under the transcriptional control of an EP procyclin promoter. Both cell lines were produced without noticeable problems and were sub-cloned immediately after selection for characterisation at the genomic and protein level. First, genomic DNA was extracted from all three cell lines and PCR was performed using two combinations of primers: one for the upstream and downstream regions the *AK3* gene (marked 1 on Fig 5) and one for the *AK3* gene itself (marked 2 on Fig 5). Results confirmed the total loss of the *AK3* gene in the ∆*ak3* line and the correct integration of both drug resistance markers, as well as the expected scheme for the single knockout and for the add-back lines (Fig 5A). Western blotting showed the loss of the 45kDa band, definitely proving its identity as AK3 (Fig 5B). In the AB cell line, a band of slightly higher apparent molecular weight was detected, in agreement with the expression of AK3 fused to the Ty1 tag that adds an extra kDa to the protein (Fig 5B). This band is more abundant (1.5 ± 0.16 fold, n = 3 separate blots) compared to the wild-type situation, as expected because of the strength of the EP procyclin promoter. It should be noted that the total amount of AK1/AK2 is increased in both cell lines (Fig 5B): by 1.6 ± 0.42 fold and by 2.8 ± 0.49 fold in the ∆*ak3* and the AB cell lines, respectively (average of 3 separate blots). IFA probing for both AK and Ty1 simultaneously confirmed the loss of flagellar AK in the *∆ak3* cell line, while the AB cell line expressed a flagellar AK that was also Ty1-tagged (Fig 5C).

Unlike the inducible *AK1-3* ^*RNAi*^ cell lines (in the 29–13 background)[10], the *∆ak3* cell lines exhibit a slight but significant growth phenotype in culture (Fig 5D). In the strain EATRO1125.T7T (procyclic stage), RNAi knockdown of all three arginine kinases induced a growth defect that was more prominent when the cells were exposed to oxidative stress [14]. Surprisingly, the AB strain resulted in slower growth (doubling time 32.7 ± 8.0 h) compared to both parental (13.9 ± 0.7 h) and *∆ak3* (16.7 ± 1.1 h) cell lines (Fig 5D). Despite slow growth in the *∆ak3* and AB cell lines, analysis by DAPI staining revealed no significant differences in the proportion of 1K1N, 2K1N and 2K2N cells between all three cell lines (Fig 5E). This suggests that the growth impairment is not associated with any particular cell cycle stage. No striking modifications of cell shape were observed.

**Fig 6:**
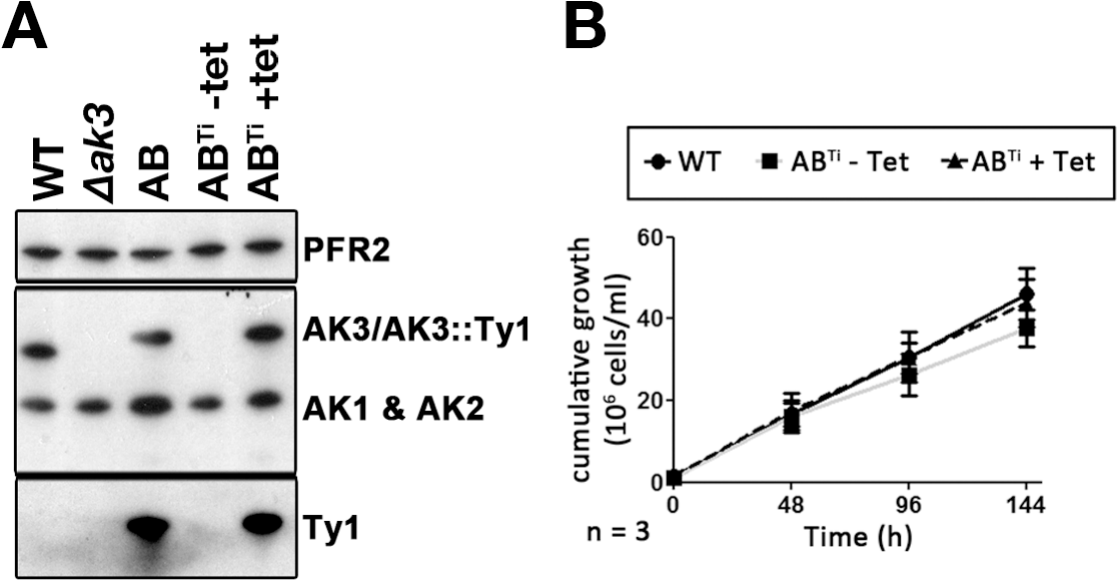
Conditional expression of AK3::Ty1. **A.** Western blotting with the Paris anti-TcAK antiserum, stripped and probed with the BB2 antibody and the anti-PFR2 as loading control shows absence of AK3 in the *∆ak3* cell line, with expression of AK3 restored with AK3::Ty1 in the AB cell line. The AB^Ti^ cell line expresses AK3::Ty1 exclusively with tetracycline-induction. **B.** The AB^Ti^ cell line exhibits a slight growth impairment when grown in the absence of tetracycline (and so in the absence of AK3::Ty1) that is not statistically significant compared to WT cells or to the tetracycline-induced AB^Ti^ cells.

To further understand the significance of these unexpected results, the AB cell line was transfected with the pHD360 vector that constitutively expresses the tet-repressor [27]. This generated the AB^Ti^ cell line where AK3::Ty1 expression should be inhibited by the tet-repressor in the absence of tetracycline but should be restored in its presence [29]. Western blot analysis with the anti-TcAK or with the anti-Ty1 confirmed AK3::Ty1 expression to be strictly dependent on the presence of tetracycline (Fig 6A). The level of AK3::Ty1 appeared to be comparable to that of the original AB cell line. However, the fully induced AB^Ti^ differed from the AB in that the expression of AK1 and AK2 was not simultaneously increased (Fig 6C). Growth curves generated for the AB^Ti^ cell line with or without tetracycline induction revealed that the non-induced AB^Ti^ cell line had a slightly impaired growth rate (15.1 ± 2.0 h) but this was not statistically significant. AB^Ti^ cells induced with tetracycline for 24 h prior to starting the growth assay negated this growth phenotype, reverting to growth rates close (14.8 ± 1.0 h) to that of the WT cell line (Fig 6B). Taken together, we hypothesize that the impaired growth observed in the AB cell line may be related to the overexpression of all 3 AKs, a phenotype not observed in the AB^Ti^ cell line. It should be noted that overexpression of an epitope tagged version of AK1 was lethal in the bloodstream stage of *T*. *brucei*, showing that perturbation of AK protein could have deleterious effects [14].

### ∆*ak3* cell line has reduced motility unrelated to structural defects in the flagellum

**Fig 7:**
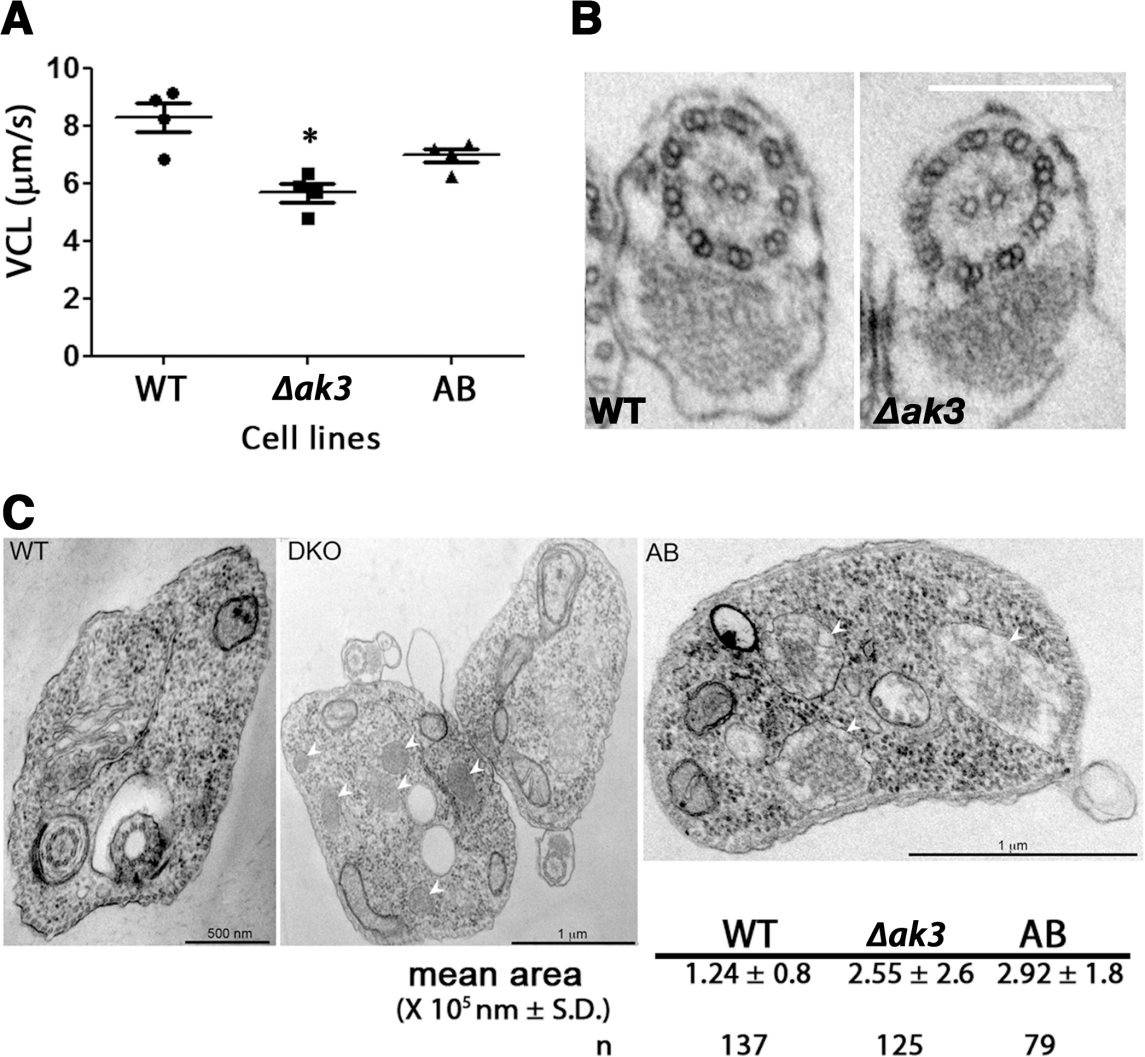
Loss of AK3 reduces swim velocity without visible alteration of flagellar morphology. A. *∆ak3* cells show a significantly reduced VCL (Velocity, CurviLinear) compared to WT cells (P = 0.005). This impairment in swim speed was partially restored in the AB cell line where VCL was significantly higher than that of *∆ak3* cells (P = 0.02) but not equivalent to that of WT cells. Each data point represents an independent experiments with more than 200 tracks analysed for each cell line. B. The reduced VCL for the *∆ak3* cell line was not related to visible defects in the axoneme structure of the flagellum. Transmission electron microscopy images of WT (n = 33) and *∆AK3* (n = 22) cells were analysed and no visible disruption in neither the 9 + 2 doublet conformation nor the paraflagellar rod were detected in all images. The scale bar represents 200 nm. C. Transmission electron microscopy images of *∆ak3* cells revealed an increase in the number and the size of visible glycosomes (white arrows) compared to WT cells, and the total area occupied by glycosomes was twice that observed in WT cells. This phenotype was not reverted in AB cells.

Knockout of *AK3* resulted in a slight but statistically significant (P < 0.05) reduction in motility compared to WT cells (Fig 7A). The VCL (Velocity, CurviLinear) was measured at cell densities of 6–9.10^6^ cells/ml as this was the culture density used for tsetse infection experiments. This reduced motility was partially restored in the AB cell line. To evaluate the consequences of absence of AK3 in the knockout cell line and the possible negative effect of overexpression of AK1/AK2, transmission electron microscopy analysis was carried out. Attention was initially focused on the flagellum but no visible structural defects could be detected neither in the 9 + 2 microtubule doublets of the axoneme and its outer dynein arms, nor in the paraflagellar rod and the membrane in transmission electron microscopy images of the WT (n = 33) and ∆*ak3* (n = 22) cell lines (Fig 7B). Curiously, examination of these images revealed an increase in the size and in the number of parasite glycosomes of ∆*ak3* and AB cell lines (Fig 7C). This would suggest that disruption of the endogenous flagellar AK in *T*. *brucei* resulted in changes to the glycosomes, a phenotype that was not reverted to that of WT in AB cells expressing the exogenous TbAK3::Ty1. This unexpected phenotype could be related to the modified expression of AK3 or to the overexpression of AK1/AK2, especially since AK2 has been shown to be present in glycosomes [14,43].

### AK3 confers a competitive advantage in tsetse infections

We next attempted to determine if absence of AK3 had an effect on tsetse infection. Because of the severe reduction in growth in the AB cell line, initial experiments were carried out and tsetse flies were dissected at 10 days post infection, revealing comparable infection rates for *∆ak3* and AB cell lines, albeit both lower than that of tsetse infected with WT cells (data not shown). We reasoned that the disparity in doubling times may be interfering with any affect AK3 absence/presence may have on tsetse infection rates, and proceeded to infect and score for parasite presence at 20 days or more post infection.

**Fig 8:**
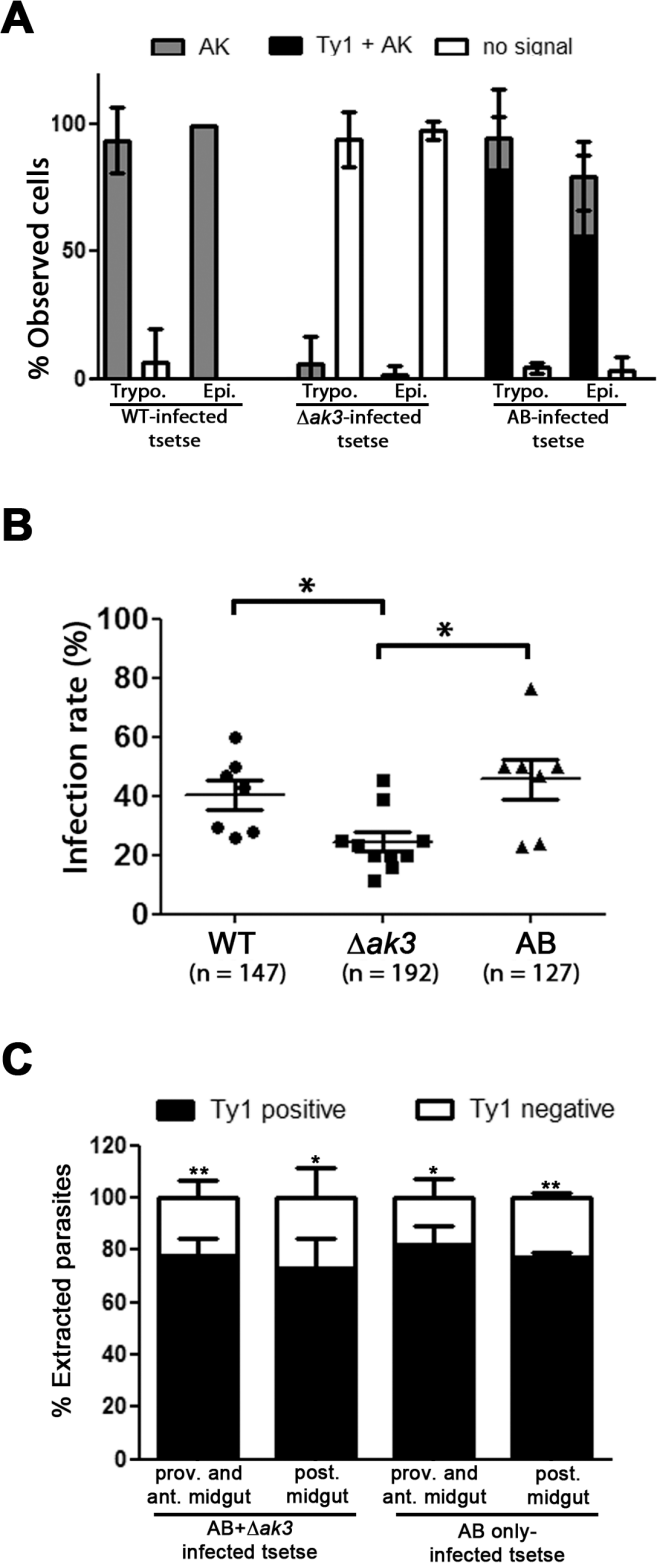
Absence of AK3 causes reduced infectivity and competitiveness in tsetse flies. A. IFA carried out on parasites extracted from tsetse flies infected with WT, *∆ak3* or AB cells indicate that the majority of parasites maintain their transgenic status even after more than 21 days in the tsetse, with the majority of WT cells positive for flagellar AK signal but not Ty1 signal, the majority of *∆ak3* cells negative for both flagellar AK and flagellar Ty1 signal, and the majority of AB cells positive for both AK and Ty1 signal. This was observed in both trypo- and epimastigote cells. The graph represents data from 3 independent experiments from harvest of the proventriculus, anterior midgut and the posterior midgut with n = total number of cells observed. B. *∆ak3* cells have a significantly lower rate of infection (24.5 ± 10.3%) towards tsetse compared to WT (40.4 ± 13.0%) and AB cells (45.6 ± 18.2%). Each data point represents an independent experiment with n = total number of dissected flies. C. When a mixed population of AB and *∆ak3* cells were used to infect flies, the majority of parasites obtained from dissected flies were positive for Ty1 as determined by IFA. This was the case for parasites extracted from the posterior midgut (n = 302) or the proventriculus and anterior midgut (n = 306).

Immunofluorescence assays with simultaneous probing for AK (anti-TcAK) and AK3::Ty1 (anti-Ty1) in trypanosomes extracted from tsetse in infection experiments revealed that no significant genetic drift occurred in the parasite population during the 20 day incubation period leading up to dissection (Fig 8A). The majority of both trypomastigotes and epimastigotes extracted from tsetse infected with WT cells were only positive for a flagellar AK signal, while the majority of parasites taken from *∆ak3*-infected tsetse had neither AK nor Ty1 signal in their flagellum. The majority of parasites extracted from AB-infected tsetse had both an AK signal and a Ty1 signal in their flagellum as expected. Infection with the WT strain resulted in the classic frequency of flies presenting an infection in their midgut (40.4 ± 13.0%). From the same infection experiments, it was observed that tsetse infected with *∆ak3* cells were less likely to be infected when dissected at more than 20 days post infection (Fig 8B), with a significantly lower rate of infection (24.5 ± 10.3%). Flies infected with AB trypanosomes had a higher rate of infection (45.6 ± 18.2%) than tsetse infected with *∆ak3* cells and similar to WT values, suggesting that AK3 confers an advantage to trypanosomes in the context of tsetse infection. This result was reproduced in 10 independent experiments (4 comparing WT, *∆ak3* and AB, 3 comparing WT and AB, 3 comparing *∆ak3* and AB). The infection rates shown here are for midgut infections only, as infections in the salivary glands were sporadic and low regardless of the cell line and no trend could be discerned from this (data not shown). Infections with the AB^Ti^ cell line were also attempted but the presence of tetracycline increased significantly the mortality of flies and hindered data interpretation (data not shown).

As tsetse infection experiments showed that the absence of AK3 resulted in reduced infectivity by procyclic trypanosomes, we next questioned if AK3 positive cells would have a competitive advantage against AK3 negative parasites when infecting flies. To address this, we co-infected tsetse with an equal concentration of *∆ak3* and AB parasites and carried out an IFA on parasites extracted from the posterior midgut and anterior midgut. The majority of parasites extracted in these co-infection experiments had a Ty1 signal in the flagellum (Fig 8C), indicating that the population of parasites infecting the experimental tsetse were AB cells. Taken together, these data further support that the presence of AK3 confers an advantage to trypanosomes during fly infection.

## Discussion

### Flagellar targeting of AK3 and related proteins

It has previously been documented that trypanosomatid AKs have remarkable similarity with the AK of arthropods, suggesting a putative horizontal gene transfer event occurring in their ancestor from a common arthropod host [37,44]. Our phylogenetic analysis suggests that this horizontal transfer occurred much earlier in the evolution of arthropods from a yet unidentified ancient host, and therefore prior to adaptation for life within the tsetse fly. Moreover, more recent species-specific gene duplications allowed the emergence of specialized AKs independently in both *T*. *congolense* and *T*. *brucei*. Our fractionation experiment shows that out of the three endogenous *T*. *brucei* AKs, only AK3 localises to the flagellum. This confirms recent data obtained upon over-expression of epitope-tagged AK versions that showed a flagellar localisation for AK3 whereas AK1 and AK2 are found in the cytosol and in glycosomes respectively [14]. The flagellar targeting is conferred by the unique amino-terminal extension that is necessary and sufficient to send a GFP reporter to the flagellum [14].

Here, we propose that AK3 flagellar targeting is achieved by its N-terminal extension that shares remarkable similarities with the flagellum calcium-binding protein (FCaBP) in *T*. *cruzi* or the calflagins in *T*. *brucei*. These acidic proteins (pI ~4.5) possess an amino-terminal tail of 24 amino acids that is necessary and sufficient for targeting to the flagellum [38,39]. First, the protein is acylated in positions 2 (myristoylation) and 3 or 4 (palmitoylation), probably to allow membrane association [38,40]. However, this is not sufficient to confer flagellar location. Analysis of the crystal structure of FCaBP revealed the presence of a surface patch of positive residues (mostly lysines)[41] that turned out to be essential for protein location to the flagellum [39]. Like for calflagins, masking that sequence with an epitope tag prevents AK3 flagellum targeting [14]. Moreover, AK3 is missing from *Leishmania*, which is also the case of calflagins. Unfortunately, the crystal structure is only known for the *T*. *cruzi* AK [45] that does not localise to the flagellum and it is therefore not possible at present to know the structuration and exposure of the amino-terminal domain of AK3. Finally, these sequences are different from the ones used to target structural proteins to the paraflagellar rod or the axoneme.

### Role of AK3 in *T*. *brucei*

Amongst African trypanosomes, *T*. *brucei* and *T*. *congolense* have the most complicated life cycle within the tsetse [4]. Upon ingestion, stumpy forms differentiate into procyclic cells, establishing an infective population within the tsetse midgut before migrating anteriorly towards the foregut and mouthparts for *T*. *congolense*, whereas *T*. *brucei* needs to reach the salivary glands to undergo another round of differentiation, proliferation and ultimately production of metacyclic trypanosomes [46]. Survival in the midgut and migration anteriorly towards either the salivary glands or the proboscis of the tsetse would therefore be challenges common to both parasite species.

AK3 was found to be more highly expressed in procyclic trypanosomes compared to their bloodstream counterparts based on observations using IFA and western blots. This is in agreement with previous findings on the mRNA level using northern blots [14]. As RNA levels do not always correspond to levels of expression [47,48], our data suggests that the relatively higher *AK3* mRNA levels in the procyclic form is in agreement with an increase in protein expression. This change in expression may reflect the different metabolism of these life cycle stages [49,50] or correspond to specific additional requirements during midgut infection.

What could be the function of AK3 in the flagellum? This organelle is central to the parasite cycle by controlling cell morphogenesis [31,51], cell division [52–55] and adhesion to the epithelium of the salivary gland [56]. The *∆ak3* cell line showed a slight impairment in growth rate *in vitro* that was further exacerbated in the AB cell line. However, no statistically significant difference in growth rate could be observed in the AB^Ti^ cell line, suggesting that absence of AK3 is not essential *per se* in culture conditions for the procyclic form. The only visible phenotype was a slight reduction in motility, without noticeable impairment in base-to-tip or tip-to-base beating of the flagellum. This is likely to be insufficient to perturb cell division, as reported for various RNAi mutants where flagellum motility is reduced but not blocked [57,58].

The situation could be different *in vivo* where parasites need to migrate from the posterior to the anterior midgut. A previous investigation where parasite forward motility was ablated by suppression of the *DNAI1* gene demonstrated that this prevents the migration of trypanosomes from the posterior to the anterior midgut of the tsetse [13]. Here, a reduction in the frequency of infected flies was observed that was compensated in the AB cell line, indicating that presence of AK3 in the flagellum, albeit not essential, would confer an advantage. This finding is supported by competition experiments revealing a bias in favour of the AB cell line for infecting tsetse flies. These results could be put in parallel with the tsetse infection phenotype observed in procyclin-null mutants that can still complete the infection cycle within the tsetse, albeit at a lower frequency but that lose a degree of competitiveness compared to control cell lines [59]. Overall, this suggests that a degree of selection pressure is present against parasites that do not express AK3 during midgut infection.

We propose that this slight advantage could result from the functioning of AK as phosphagen kinases that are involved in ATP buffering. For example, creatine kinase is localised in the flagellum of sea urchin sperm to mediate regeneration of ATP during periods of high activity [16,17]. Little is known so far about access of ATP to the flagellum and control of its concentration, but it is likely that multiple systems function together to ensure that sufficient amounts of ATP are present for flagellum motility but also for other processes, such as intraflagellar transport, the movement of large protein complexes between microtubule doublets and the flagellum membrane [60]. For example, two adenylate kinases (the enzyme that ensures the interconversion of 2 ADP in ATP and AMP) are associated to the paraflagellar rod of the flagellum via the presence of specific targeting sequences. However, RNAi knockdown failed to reveal a specific function in culture [61]. In this context, one could imagine that flagellar AK activity could contribute to the infection during key transition stages of the life cycle. Transferring trypanosomes to a buffer deprived of energy sources such as PBS resulted in a rapid arrest of flagellum motility. This drop was observed with equal kinetics in control cells or in parasites deprived of AK3 (our unpublished data), indicating that this buffering system is not able to cope with sharp changes in energy supply, but could be involved in more subtle variations. AK3 could possibly perform other functions, such as the transfer of signalling molecules from the flagellum to the cell body [62], what could be significant in trypanosomes whose flagellum is attached to the cell body by a sophisticated adhesion system [63–67], that is actively remodelled during parasite development in the tsetse fly [31]. A more remote option could be an interaction with the flagellar skeleton, for example for a direct regulation of dynein arm activity. Intriguingly, arginine kinase has been reported to interact with actin in scallop muscles [68] and actin has been found in most eukaryotic flagella where it could participate to the control of dynein arm activity [69].

In the field, mixed trypanosome infections in tsetse are not unusual, whether it is between different trypanosome species or between different subspecies. Molecular identification of trypanosome species from field-caught *Glossina pallidipes* in Tanzania identified up to 40% of dissected fly proboscises to harbour more than one species of trypanosome, while a similar study on *Glossina palpalis* in Cote d’Ivoire identified mixed infections of *T*. *congolense*, *T*. *vivax* and *T*. *brucei* in field-caught flies [70,71]. Laboratory infections of *G*. *m*. *morsitans* have demonstrated that flies with a mature parasite infection are just as susceptible to infection by a second trypanosome species compared to uninfected flies [72]. Overall, the fitness benefit conferred by flagellar AK could be responsible for its presence in African trypanosomes that initiate their cyclical development with a midgut stage.
